# Exploring molecular backgrounds of quality traits in rice by predictive models based on high-coverage metabolomics

**DOI:** 10.1186/1752-0509-5-176

**Published:** 2011-10-28

**Authors:** Henning Redestig, Miyako Kusano, Kaworu Ebana, Makoto Kobayashi, Akira Oikawa, Yozo Okazaki, Fumio Matsuda, Masanori Arita, Naoko Fujita, Kazuki Saito

**Affiliations:** 1 RIKEN Plant Science Center, Tsurumi-ku, Suehiro-cho, 1-7-22 Yokohama, Kanagawa, 230-0045, Japan; 2 Current Address: Bayer CropScience N.V., Technologiepark 38, 9052 Gent, Belgium; 3 National Institute of Agrobiological Sciences 2-1-2 Kannondai, Tsukuba, Ibaraki 305-8602, Japan; 4 Kobe University Organization of Advanced Sciences and Technology 1-1 Rokkodaicho, Nada-ku, Kobe, 657-8501, Japan; 5 Department of Biophysics and Biochemistry, The University of Tokyo, Bunkyo-ku Hongo 7-3-1, Science Bldg 3, 113-0033 Tokyo, Japan; 6 Faculty of Bioresource Sciences, Akita Prefectural University, Akita city, Akita, 010-0195, Japan; 7 Graduate School of Pharmaceutical Sciences, Chiba University, Inohana 1-8-1, Chuo-ku, Chiba, 260-8675 Japan

## Abstract

**Background:**

Increasing awareness of limitations to natural resources has set high expectations for plant science to deliver efficient crops with increased yields, improved stress tolerance, and tailored composition. Collections of representative varieties are a valuable resource for compiling broad breeding germplasms that can satisfy these diverse needs.

**Results:**

Here we show that the untargeted high-coverage metabolomic characterization of such core collections is a powerful approach for studying the molecular backgrounds of quality traits and for constructing predictive metabolome-trait models. We profiled the metabolic composition of kernels from field-grown plants of the rice diversity research set using 4 complementary analytical platforms. We found that the metabolite profiles were correlated with both the overall population structure and fine-grained genetic diversity. Multivariate regression analysis showed that 10 of the 17 studied quality traits could be predicted from the metabolic composition independently of the population structure. Furthermore, the model of amylose ratio could be validated using external varieties grown in an independent experiment.

**Conclusions:**

Our results demonstrate the utility of metabolomics for linking traits with quantitative molecular data. This opens up new opportunities for trait prediction and construction of tailored germplasms to support modern plant breeding.

## Background

Modern crop breeding techniques such as wide crossing and marker-assisted selection have been highly successful in improving the quality traits of rice [1,2]. However, as slow selection processes and narrow germplasms [3] have raised doubts on how much further current strategies can take us [4], we must diversify the used genetic material and develop novel breeding technologies.

While the germplasm that is actively used for rice breeding may be narrow, the total number of rice varieties is enormous due to its very long domestication history [5]. The broader use of available genetic variance has great potential, both to improve crops directly [6] and to elucidate molecular determinants behind quality traits (see e.g. [7]). Unfortunately, the necessary molecular characterization is often prohibitively expensive for large seed collections.

Genetic core collections of relatively small size have been developed in several rice genebanks to obtain manageable but still representative selections, e.g., the Rice Germplasm Core Set (RGCS) from the International Rice Research Institute (623 accessions) [8], the GCore collections (16 × ~120 accessions) [9], the EMBRAPA Rice Core Collection (ERiCC, 242 accessions) [10] and the rice diversity research set (RDRS) [3]. Of these, the RDRS is particularly interesting because its restriction fragment length polymorphism (RFLP) marker diversity is highly representative of cultivated rice (*Oryza sativa *L.); yet with only 67 varieties, it is small enough to allow comprehensive molecular profiling.

Direct relationships between metabolic composition and genotype and phenotype have been shown for the model plant *Arabidopsis thaliana *using both recombinant inbred lines [11] and natural varieties [12,13]. Metabolomics has emerged a key technology for characterizing crop germplasms; it has the potential to provide a breakdown of complex high-level traits by expressing them as a sum of correlated quantitative molecular features. Such molecular factorization may increase the physiological understanding of quality traits and provide clues for possible implications associated with selecting for them. This is highly relevant since metabolic composition is itself an important quality trait as it is tightly connected to the taste and the nutritional and physical characteristics of the harvested material [14].

With these considerations in mind, we aimed to (i) chart the metabolic diversity of kernels from the RDRS and (ii) investigate the covariance between metabolite profiles and quantitative quality traits. A previous study of 18 of the RDRS varieties using ^1^H-NMR did not reveal any relationship between metabolomic and overall genetic diversity [15]. As this finding may be attributable to the small sample size and insufficient resolution of the applied technique, we aimed to obtain metabolomic coverage as high as possible and decided to profile the complete RDRS. Because no current single technology can separate all compounds equally well [16], we chose to integrate data from 4 complementary mass spectrometry (MS) -based platforms, and thereby reducing bias towards any particular chemical subclass of metabolites [17]. The resulting data showed clear compositional differences among the 3 genetic subtypes Indica I, Indica II and Japonica. Using a novel extension of orthogonal projection to latent structures (OPLS) [18] that facilitates the handling of multi-block data (MB-OPLS), we found that given the metabolic composition, 10 of the 17 studied traits, including the important kernel size [19], ear emergence day [20], and amylose ratio (abundance amylose/total starch content), could be predicted indicating robust trait-metabolite covariance.

Starch composition is a major determinant of the taste and texture of cooked rice [21]. The packing characteristics of starch also determine the proportion of desired translucent kernels to kernels with chalky white cores that are prone to breakage during processing [22]. Our metabolomics model confirmed previously observed strong negative associations between fatty acids/lipids and amylose ratios [23,24]. Furthermore, the same model accurately predicted the amylose ratio for an independent set of varieties grown in a remote field. However, starch synthase IIIa knock-out lines (*ssIIIa*) with white-core phenotypes had very high amylose ratios without the accompanying expected fatty acid/lipid composition, suggesting an important role of fatty acids in starch packing. Taken together, our results demonstrate the usefulness of metabolomic profiling of genetically diverse varieties for linking quality traits with molecular features.

## Results

### Multi-platform metabolomics of the RDRS

**Figure 1:**
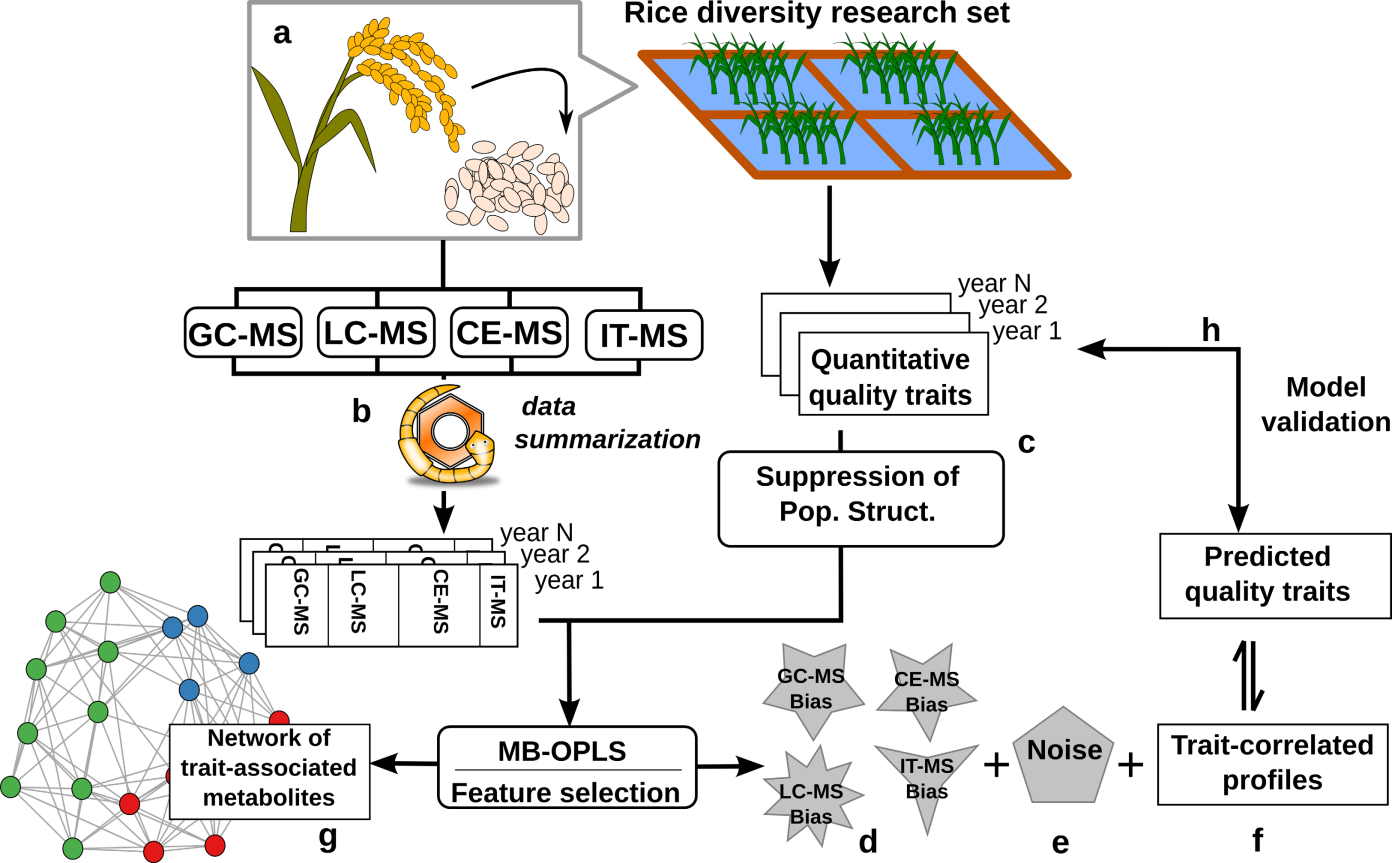
**Metabolomics characterization of the RDRS**. Seeds were collected from field-grown rice and analyzed on 4 metabolomics platforms (a). Multi-platform metabolite profiles were summarized to obtain non-redundant data (b). Quantitative quality trait data were gathered and pre-treated to remove the correlation with genetic population structure (c). MB-OPLS was used to decompose the metabolite profiles to platform-specific systematic bias (d), noise (e) and the trait-correlated variance used for predicting each trait (f). A novel feature selection method was used to identify trait-associated metabolites that were used to generate network visualization (g). Cross-validation and an independent experiment were performed to validate the derived models (h).

Rice plants from the 67 RDRS varieties plus Nipponbare (reference Japonica variety), Kasalath (reference Indica variety), and the Pokkari variety were grown in a field in Tsukuba in 2005 and harvested after maturation [25]. Brown rice kernels were ground and analyzed in parallel using 4 MS-coupled platforms, i.e. gas chromatography-(GC) time-of-flight (TOF)-MS (GC-MS) for smaller compounds, liquid chromatography-quadrupole-TOF-MS (LC-q-TOF-MS) for large hydrophilic compounds, ion trap-TOF-MS (IT-MS) for polar lipids [26] and capillary electrophoresis-TOF-MS (CE-MS) for ionic compounds (Figure 1). The resulting data were pre-processed, normalized [27] and summarized [17,28] (see Additional File 1, Supplementary Methods). Metabolite abundances were determined for 156 distinct metabolites and 1496 unknown analytes (Additional File 2, Supplementary Data 1). Principal component analysis (PCA) of predicted metabolite physicochemical properties indicated that the detected metabolites covered 87% of the chemical diversity of the metabolites listed in RiceCyc (Additional File 1, Figure S1). Reference data for 17 quality traits (Additional File 1, Table S1) were collected from previous analyses and the National Institute of Agrobiological Sciences (NIAS) genebank [29].

**Figure 2:**
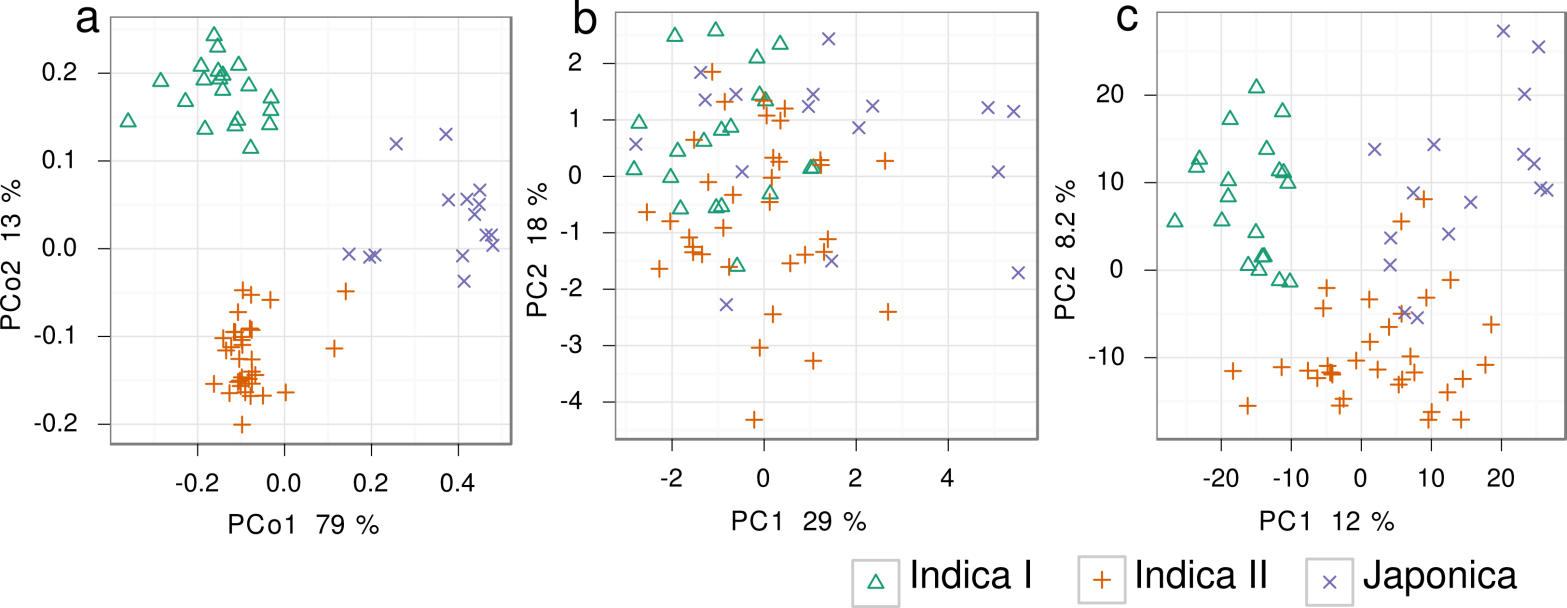
**Genetic subtypes in 3 spaces**. (a) Principal coordinates analysis of the genetic distances between the varieties indicate the presence of 3 major sub-populations, Indica I (20 varieties), Indica II (34 varieties) and Japonica (16 varieties). (b) PCA of the 17 quantitative traits; (c) PCA of the complete summarized metabolite profile dataset with a total of 1652 peaks. Percentages indicate the ratio of explained to total variance.

Examining the genetic population structure of the RDRS using principal coordinates analysis on the matching coefficient-based genetic distance matrix (Figure 2a) and the STRUCTURE program (v 2.3.2.1) [30], we confirmed the presence of 3 major subtypes are Indica I, Indica II and Japonica type rice (Additional File 1, Figure S2). PCA showed that these subtypes also are distinguishable among the investigated quality traits as well as the metabolite profiles (Figure 2b, c), indicating a distinct influence of the genetic background on the visible phenotype and the metabolic composition.

**Figure 3:**
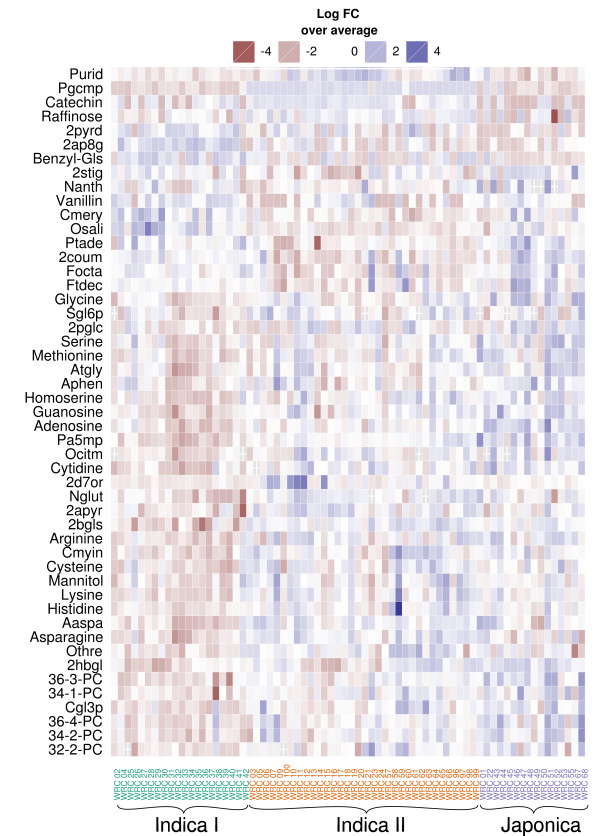
**Metabolomic heatmap of the RDRS**. Shown are the annotated metabolites that were differentially abundant among the 3 subtypes Indica I, Indica II and Japonica at a minimum 2-fold change from the average and FDR < 0.01 (Student's *t*-test.). Abbreviations defined in Additional File 2, Supplementary Data 1.

Using analysis of variance (ANOVA) to extract the metabolites that were differentially abundant among the different subtypes we noted that Indica I was characterized by a relatively low abundance of several metabolites including most amino acids and 5 of the detected phosphatidylcholines (Figure 3). Indica II and Japonica were more similar to each other, differing mainly in the contents of a few of the secondary metabolites such as catechin and trans-4-coumaric acid. With respect to the investigated quality traits, the subtypes exhibited morphological differences; Indica I- were more narrow overall than Japonica kernels and Indica II- longer than Indica I kernels (Additional File 1, Figure S3a)

### Metabolite profiles show a fine-grained correlation with genetic variation

Our results show a substantial overlap between metabolite profiles and the underlying genetic backgrounds (Figure 2c). Although of interest for comparing subtypes, this type of large-scale correlation between genotype and phenotype (metabotype) is obstructive when searching for functional associations with high-level traits [31]. Using the Mantel test [32] with 10,000 permutations, we examined whether the Euclidean distances in metabolite space between different varieties were correlated with their corresponding genetic distances both for the whole RDRS, and for the 3 subtypes separately. As expected, the highest significance was observed for the whole dataset (*P *= 0.0001) but Japonica (*P *= 0.0047), Indica I (*P *= 0.0064), and Indica II (*P *= 0.0001) were also significant on their own, indicating the presence of a fine-grained correlation between genetic diversity and metabolite abundances (Additional File 1, Figure S4).

### MB-OPLS regression predicts quality traits from metabolic composition

Before investigating trait-metabolite correlations we removed the covariance between the trait data and the population membership *Q*-matrix from the STRUCTURE program by means of multiple linear regression. As confirmed by PCA, the resulting data showed no clustering of the 3 subtypes (Additional File 1, Figure S3). Furthermore, the pre-processed traits exhibited highly individual variations, except for kernel size-weight and hull- and kernel width (Additional File 1, Figure S5).

**Figure 4:**
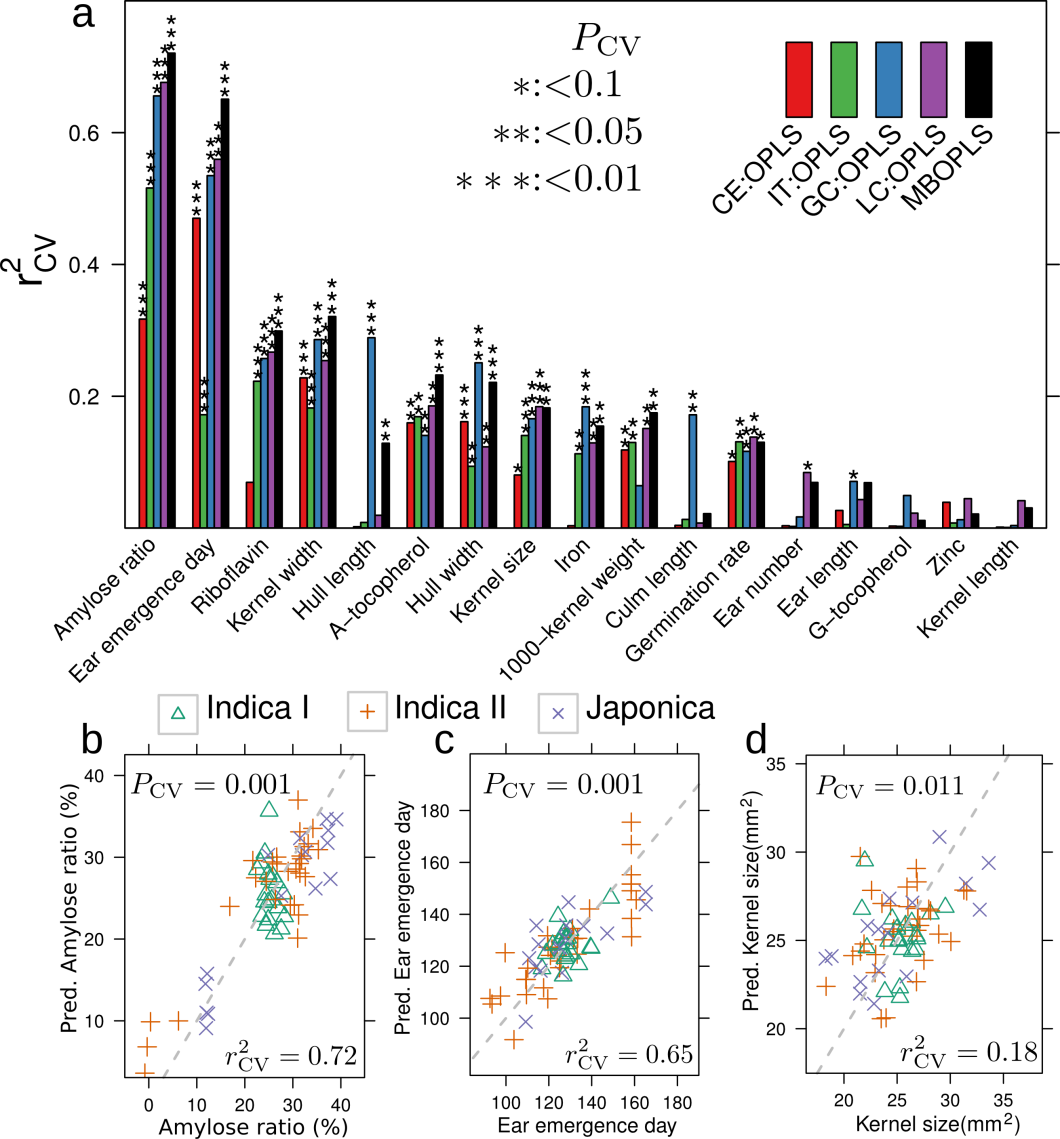
**Predicting quality traits from metabolomic composition**. (a) The predictive performance of models based on single datasets using OPLS and all datasets together using MB-OPLS. Cross-validation based $r_{\text{CV}}^{2}$ statistics equals 1 for perfect predictions. The stars indicate significance level as estimated by the empirical *P*_CV_-value. (b-d) Prediction performance during the median cross-validation run. Grey lines indicate identity.

While yielding a good metabolomic coverage (Additional File 1, Figure S1), multi-platform data may, even after normalization, contain platform-specific biases that have adverse effects on data integration methods. MB-OPLS was designed to overcome this problem by using the notion that OPLS also can be used for normalization purposes [33]. We estimated MB-OPLS models for each of the 17 traits and diagnosed their predictive performance using the squared correlation coefficient between the true and the seven-fold cross-validation (CV) predicted trait data, $r_{\text{CV}}^{2}$ (Figure 4a). We furthermore calculated the empirical *P*-value *P*_CV _that assesses the probability of observing an equal or higher $r_{\text{CV}}^{2}$ given randomized data. For comparison, we also used the original OPLS approach on each of the 4 data blocks alone. Overall, MB-OPLS performed better than any of the single platforms and predicted 10 of the 17 traits significantly well (*P*_CV _< 0.05). In particular, the models of amylose ratio and ear emergence day were remarkably accurate with $r_{\text{CV}}^{2}=0.72$ and $r_{\text{CV}}^{2}=0.65$, respectively. Other traits exhibited less reliable but still clearly significant predictions, indicating the existence of subtle but robust trait-metabolite associations. Given the strong prediction performance of the models for amylose ratio and ear emergence day, and the high agricultural interest in kernel size, we chose to examine these models more closely (Figure 4b-d).

The OPLS regression framework, and therefore also MB-OPLS, provide correlation loadings, *P_C_*, that can be used to interpret the relevance of each metabolite for the corresponding prediction. However, this value does not assign any statistical significance in terms of comparison with a postulated null-hypothesis (no trait-metabolite associations) and the variance of the observed sampling distribution of *P_C_*. To address this problem we define a probabilistic statistic for feature selection, log *B*; it scores how many times more likely the alternative hypothesis is over the null-hypothesis.

When screening for trait-associated metabolites we used both the model-based log *B *statistic and the nominal Spearman's correlation, *ρ_S_*, as a complementary bivariate method. We extracted the annotated metabolites with log *B >*0 and *ρ_S _*with an associated false discovery rate (FDR) less than 0.05. We visualized the correlation loadings for all annotated metabolites as word clouds, and listed the top 10 selected metabolites in Additional file 3, Table 1. The model for amylose ratio is characterized by high negative loadings for several fatty acids as well as choline and putrescine. For ear emergence day, tryptophan and putrescine have large positive loadings. Succinate, glucose-6-phosphate, and glycine are all positively correlated with kernel size whereas 3 lipids (18:1-lysophosphatidyl cholines (lysoPC), 18:2-lysoPC and 14:0-lysoPC) are negatively correlated. A complete list of trait-metabolite associations in given Additional File 2, Supplementary Data 2.

**Figure 5:**
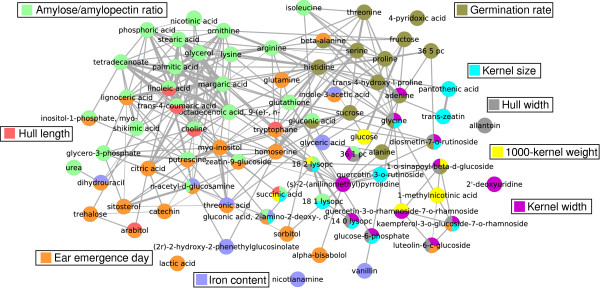
**Correlation network of the trait-associated metabolites**. The color of the nodes indicates the trait with which they are associated. Thickness of the edges indicates strength of correlation.

To obtain an overview of the trait-metabolite correlations we constructed a correlation network of the metabolites (significance of metabolite-metabolite Spearman's correlation *P <*0.001) for the 10 significant models and the germination rate since this trait had border-line significance with *P*_CV _*<*0.1 for all 4 independent datasets. The resulting graph (Figure 5) highlights the strong internal correlations of the fatty acids as well as the high overlap between the metabolites used for the morphological traits (1000-kernel weight, -size, -width and hull width, but not hull length). Several metabolites, like putrescine, are used for the prediction of more than one trait even in cases where the traits themselves are not correlated (Additional File 1, Figure S6).

### Independent experiment demonstrates robustness of the model of amylose ratio

**Figure 6:**
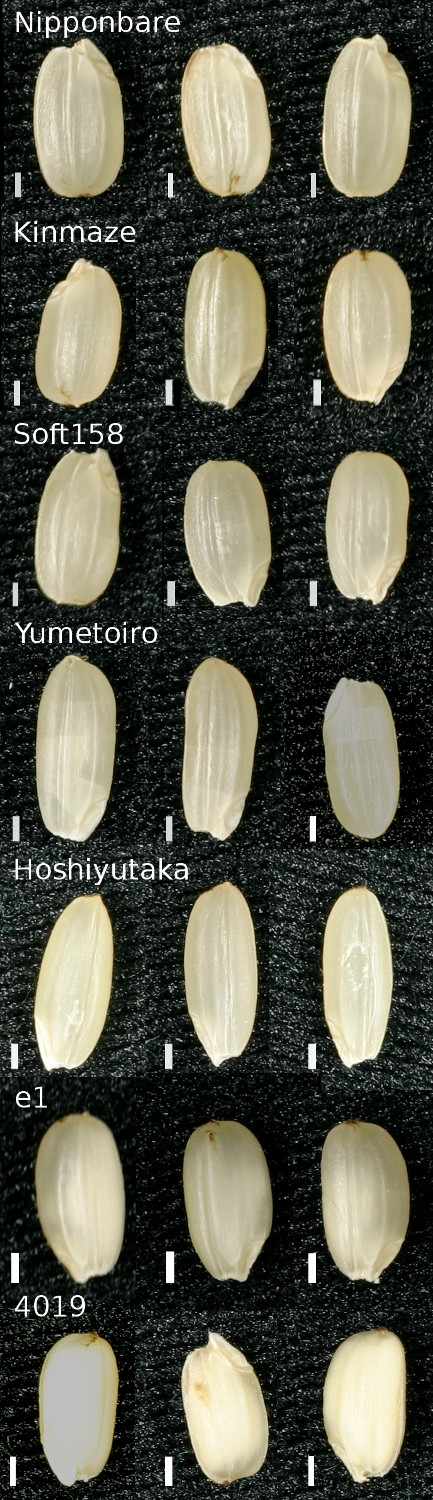
**De-hulled kernels from the varieties outside the RDRS and the two mutants *e1 *and *4019 *used in the follow up experiment**. Each variety is represented a row with kernels from three biological replicates. Overall, the natural cultivars (first five rows) have a translucent phenotype whereas among the mutants *e1 *has a white core and *4019 *is almost completely opaque. The white scale-bar indicates 1 mm.

**Figure 7:**
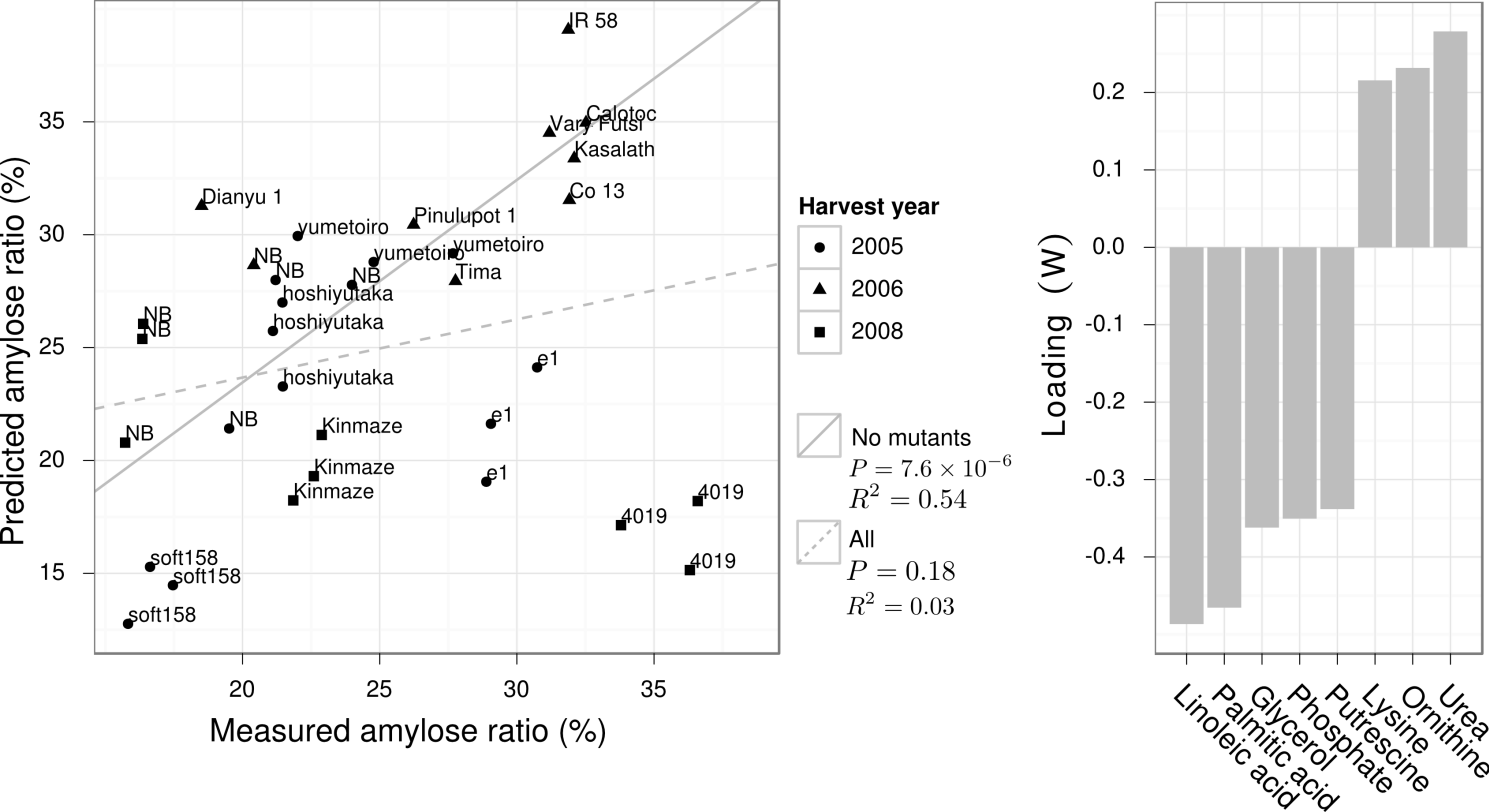
**Prediction of amylose ratio for independent samples using a model trained on RDRS data**. (a) Scatter plots of predicted and measured amylose ratio for the 4 external varieties (Yumetoiro, Hoshiyutaka, Kinmaze, Soft158) and samples from 9 representative varieties of the RDRS (Nipponbare [NB], Kasalath, IR 58, Co 13, Vary Futsi, Calotoc, Pinulupot 1, Dianyu 1 and Tima) harvested in 2005 and 2006. *P*-values assess the hypothesis that the corresponding slope is zero and *R*^2 ^indicates the model-fit. (b) Barplot showing the importance of the 7 metabolites in the subsetted MB-OPLS model (*W*). Negative loading implies a negative correlation between amylose and the corresponding metabolite.

The model for amylose ratio gave very accurate predictions highlighting a tight correlation between fatty acids and starch synthesis. To confirm the robustness of this model we selected an external set of samples including rice varieties outside the RDRS with known high- (Yumetoiro, Hoshiyutaka), middle- (Kinmaze), and low amylose ratios (Soft158). Additionally, we included the 2 amylose hyper-accumulating knock-out lines (*Tos17 *retro-transposon insert) *e1*, an *ssIIIa *mutant (Nipponbare background) and the *ssIIIa*/starch branching enzyme (*be*) double mutant *4019 *(Nipponbare/Kinmaze background) [34]. Rice kernels were obtained from different harvests from northern Japan (Akita) [34]. The selected natural varieties have high variance in their amylose ratios but all have kernels translucent kernels. The *e1 *mutant manifested a white-core phenotype [34] and the morphology of the *4019 *mutant was almost completely opaque (Figure 6). The amylose ratio was assayed using iodine calorimetry (same method as used for the RDRS), and metabolite abundances were determined using GC-MS since this platform detects most of the amylose-correlated metabolites (Figure 5). We then fitted a subsetted model for the RDRS data using only the metabolites that had log *B >*0 and were also detected in the follow-up experiment. The obtained model was used to predict the amylose ratio using the new metabolite profile data (Figure 7a). Of the selected metabolites, glycerol, linoleic acid, palmitic acid, phosphate and putrescine had the highest loadings; all exhibited a negative correlation with the amylose ratio (Figure 7b). The prediction performance for the natural varieties was highly significant (*R*^2 ^= 0.52, *p *= 7.5 × 10^-6^, Figure 7a), but not for the 2 knock-out lines that had a similar or even smaller predicted amylose ratio than their background varieties.

## Discussion

We profiled the metabolomic composition of kernels from the RDRS and investigated trait-metabolite correlations by means of a multi-platform approach. Using our multi-block extension of the OPLS algorithm we found a population structure-independent correlation between metabolite abundances and 10 of the 17 examined traits. With the majority of these traits being only weakly dependent on each other (Figure 5), this indicates a rich correlation structure and high a information content in the metabolomics data. Our study thus confirms, and widely extends, the results shown for *Arabidopsis thaliana *grown under tightly controlled conditions [11,12], for an important crop species grown under field conditions.

The MB-OPLS model for amylose ratio indicated very strong negative correlations between the amylose ratio and the abundances of palmitic acid, linoleic acid, glycerol, and putrescine, and positive correlations with 18:2 and 14:0 lysoPC (Figure 4, Additional File 1, Table S1). The two prevalent forms of starch in rice is amylose and amylopectin and a high measured amylose ratio thereby indirectly indicate a low amylopectin ratio. The link between starch-bound fatty acids/lipids has already been observed in rice [23] and maize [24], on the metabolic- and gene expression level [35] the biochemical function of this connection is unclear.

The RDRS-based model was robust enough to give good predictions for kernels from external varieties from an independent experiment despite unaccounted differences between the growth times and locations (Figure 7). Interestingly, the 2 knock-out lines were exceptions to the rule of a negative correlation between amylose ratio and fatty acid content. This indicates that the retro-transposon inserts have broken the association with the metabolite composition, and that the link between amylose ratio and fatty acids is under feed-back control. Analysis of the biochemical or genetical backgrounds of these correlations was not within the scope of this study but we note that fatty acids and lipids are good starch-complexing agents and their presence influences physicochemical properties [36]. In addition, we observed strong differences in kernel phenotype between natural varieties and the two mutants (Figure 6). Grain chalkiness is a complicated trait affected by environmental changes [37] and genetic background [38]. Our results suggest that also fatty acids/lipids have an important function in modulating the texture and structural properties of the stored starch.

The model for the ear emergence day was also very accurate (Figure 4) and gave high weight to putrescine and tryptophan (Additional file 3, Table 1). Putrescine is a major amine in rice kernels [39] and has been implicated in the regulation of plant growth and development [40]. However, transgenic rice over-expressing a gene encoding a feedback-insensitive *α*-subunit of rice anthranilate synthase (OASA1D) had increased levels of tryptophan and indole-3-acetate as well as other amino acids in kernels without a significant difference in the ear emergence day [41].

For *Arabidopsis *photosynthetic tissues, it has been shown that biomass is negatively correlated with glucose-6-phosphate and succinate levels [11]. Keeping in mind that the rice kernel is a strong energy sink with very little own photosynthetic activity, it is not surprising that we instead observed a positive correlation between glucose-6-phosphate and kernel size. This result supports the general idea that energy demand during grain-filling plays an important role in determining kernel size [42]. In a brief study of metabolite abundances and kernel sizes using a collection of backcross recombinant inbred lines between Kasalath (Indica I) and Koshihikari variety (Japonica), this pattern was not visible indicating the connection is not generally visible among all genotypes (data not shown). However, detailed dissection of the genetic background of these patterns is left to a future study.

The model for iron content showed a rather low but still significant predictive performance with $r_{\text{CV}}^{2}=0.18$ and *P*_CV _= 0.024. However, nicotianamine, known to be involved in iron metabolism [43], was of the few annotated annotated metabolites with log *B >*0 (Figure 5, Additional File 2, Supplementary Data 2). These results exemplify how metabolic profiling of genetically diverse varieties can reveal functional relationships between molecular factors and important quality traits.

## Conclusion

We summarize the main conclusions as follows.

• The overlap between metabolic and genetic profiles in the RDRS was visible with respect to general subtypes (Figure 2b), and fine differences within the more homogeneous populations Indica I, Indica II and Japonica (Additional File 1, Figure S4). This shows that metabotypic- and genotypic-covariance could be detected in a field-grown collection of natural rice cultivars of relatively limited size.

• The metabolic diversity was furthermore found to be associated with 10 of the 17 studied quality traits (Figure 4) showing that trait-metabolite associations are common, and that they can be uncovered by profiling natural varieties. The resulting network of the trait-associated metabolites provided an overview of the molecular backgrounds of the traits (Figure 5) highlighting known (e.g. fatty acids and amylose ratio) and novel patterns (e.g. tryptophan and ear emergence day). From a technical point of view, we conclude that the applied metabolomics platforms were complementary and that integrating the datasets gave overall better prediction performance than achievable with data from any single platform.

• The amylose ratio model showed that trait-metabolite associations can be robust enough to allow for prediction across independent sets of cultivars grown on different occasions in remotely separated fields (Figure 7). A contributing reason for this robustness maybe that the mature kernel has little metabolic activity on its own and is less influenced by environmental factors than e.g. the leaves.

Taken together, these results show that metabolomics may be used to factorize important quality traits into distinct genotype-correlated molecular features. These features can both aid physiological interpretation and potentially be used as bridges to identify trait-(metabolite)-associated loci. This concept is similar to the current advancements in plant phenomics. There, complex high-level traits are being modeled using sets of simpler traits that have tighter relationships with genetic determinants than the high-level trait itself [44]. With metabolomics, traits can be factorized to an even higher resolution that may point directly to underlying genetically-dependent molecular determinants. As genetic data of adequate resolution are currently not available for RDRS, that analysis was not within the scope of our study. However, as such data are anticipated, the value of the dataset presented here is expected to increase.

## Methods

### Plant material

The RDRS and an external set of rice varieties as well as two knockout mutants (*e1 *and *4019*) were used for this study. Plant growth and harvesting were carried out as described in Additional File 1, Supplementary Methods.

### Metabolite profiling

All data was log_2 _transformed and scaled to unit-variance prior to further data analysis. All peaks with more than 30% missing values were excluded.

The multi-platform data was summarized by unifying metabolite identifiers to a common referencing scheme using the MetMask tool [28]. The four matrices were then concatenated and correlated peaks with the same annotation were replaced by their first principal component. Coverage of the chemical diversity was calculated as described by [17]. The summarized dataset is available at http://prime.psc.riken.jp/?action=drop_index and as Additional File 4, Supplementary Data 3. Detailed information of extraction, MS conditions and data processing of GC-MS, LC-MS, CE-MS and IT-MS were performed as described in Chemical analysis metadata in the section of Metabolomics metadata.

### Data analysis

All data analyses were performed using R v2.12.1. Network visualization was done using Cytoscape and the GOlorize plug-in [45]. Missing value robust PCA was performed using the pcaMethods package [46]. See Additional File 1, Supplementary Methods for detailed description of the data analysis.

#### Correction for population structure

Each column trait data vector, *Z_j_*, was compensated for the differences arising from the different sub-populations by setting

$$Z_{j}=QB+Y_{j}$$

where **Q **is the estimated population membership matrix from the STRUCTURE program and *B *is the vector of coefficients estimated by least-squares regression.

#### MB-OPLS

The MB-OPLS regression method consists of two steps. In the first, OPLS models of each block *i *and pre-processed trait vector *Y_j _*are formed where the *n*_samples _× *n*_peaks,*i *_metabolite data matrix, **X***_i_*, is decomposed into a *Y_j_*-correlated part, $T_{i,j}W_{i,j}^{T}$, a *Y_j_*-uncorrelated part, $T_{i,j,O}P_{i,j,O}^{T}$, and the unmodeled variance **E **as

$$X_{i}=T_{i,j}W_{i,j}^{T}+T_{i,j,O}P_{i,j,O}^{T}+E_{i,j},$$

and new regressor matrices **X**_Top,*j *_for each trait *j *are formed by concatenation:

$$X_{\text{Top},j}=\left[T_{1,j}W_{1,j}^{T}+E_{1,j};\ldots ;T_{n,j}W_{n,j}^{T}+E_{n,j}\right].$$

Top-level models are then estimated by ordinary OPLS regression between **X**_Top,*j *_and *Y_j_*. MB-OPLS for a single block is equivalent to ordinary OPLS.

Each MB-OPLS model has *j *+ 1 parameters corresponding to the number of orthogonal components (number of columns in **T**_*i*, *j*, *O*_) used for the block-, and top-level models respectively. We optimize these parameters by seven-fold internal cross-validation (CV).

The diagnostical statistic $r_{\text{CV}}^{2}$ of the complete model is estimated in an external seven-fold CV where a set of samples is held out to serve a test-set and the remaining are used to construct the internally cross-validated model. This process is repeated for each CV-segment to obtain independent predictions of the complete *Y_j_*. In order to test the significance of the model, we shuffle *Y_j _*one-thousand times, calculate $r_{\text{CV}}^{2}$, and count the number of times, *n*_0_, when $r_{\text{CV}}^{2}$ for the shuffled data is more than or equal to $r_{\text{CV}}^{2}$ for real data and form the biased *P*-value estimate *P*_CV _= (*n*_0 _+ 1)*/*(1000 + 1). This CV approach is computationally intensive and was therefore computed on in parallel using the multicore package [47]. Since the $r_{\text{CV}}^{2}$ depends on the way the samples are divided in to training and test sets, we calculate $r_{\text{CV}}^{2}$ 50 times and report the median of these runs.

#### Feature selection

We assess how informative each metabolite is in each model by estimating the density of the sampling distributions for its correlation loading, *d*(*p_C_*), by bootstrapping the regression model, and the density distribution under the null-hypothesis (**X **and *Y_j _*are independent), *d*(*p_C_|H*_0_), by randomization of *Y_j_*. We then calculate a score for the relevance for each metabolite as

$$b=\frac{d\left(p_{C}\right)\left[1-P\left(H_{0}\right)\right]}{d\left(p_{C}\right)\left[1-P\left(H_{0}\right)\right]+d\left(p_{C}|H_{0}\right)P\left(H_{0}\right)},$$

setting the *a priori *expected probability of *H*_0 _to 0.95. Our statistic log $B=\log\frac{b}{1-b}$ is then greater than zero for metabolites with loadings that are robustly larger than expected given that *H*_0 _was true.

## Authors' contributions

HR and MK analyzed the data, designed experiments and wrote the manuscript. MK performed GC-MS analysis. KE provided plant material and designed the study. AO performed CE-MS analysis. FM performed LC-MS analysis. YO performed IT-MS analysis. NF provided plant material. MA and KS conceived of and designed the study. All authors read and approved the final manuscript.

## Supplementary Material

Additional file 1 **Supplementary methods, tables metabolomics meta-data**. Click here for file

Additional file 2 **Supplementary datasets**. Click here for file

Additional file 3 **Influential metabolites**. Correlation loading, *P*_*C*_, indicate proximity between the metabolite and the trait-correlated variance. log *B *indicates how many times more likely the alternative hypothesis (actual association between trait and metabolite) is than the null-hypothesis (no association). Spearman's correlation *ρ_S _*with associated FDR indicates the direct bivariate correlation. Word clouds are ordered alphabetically and have font sizes proportional to the corresponding correlation loading (*P*_*C*_). Green and red indicate apositive and negative correlation with the trait, respectively. The spatial layout is abitrary. Where present, initial capital letters of the metabolite abbreviations indicate type of molecule (F, fatty acid; C, alcohol; P, purine/pyrimidine; S, sugar; N, nitrogen containing; A, amino acid; 2, secondary metabolite) Click here for file

Additional file 4 **The summarized metabolomics data of the RDRS**. Click here for file
