# Supplementary material for: Exploring molecular backgrounds of quality traits in rice by predictive models based on high-coverage metabolomics
Source: BMC Syst Biol. 2011 Oct 28;5:176. doi: 10.1186/1752-0509-5-176 (PMC3305925; doi:10.1186/1752-0509-5-176)
Supplement: Additional file 1 — Supplementary methods, tables metabolomics meta-data. [file 1752-0509-5-176-S1.PDF]

Supporting Information for *Predicting quality traits in cultivated rice using multi-platform metabolomics*

September 26, 2011

1 Supporting methods

1.1 MB-OPLS

The MB-OPLS regression method consists of two steps. In the first, OPLS models of each block i and pre-processed trait vector Y_j are formed where the $n_{\text{samples}} \times n_{\text{peaks},i}$ metabolite data matrix, $\mathbf{X}_i$, is decomposed into a Y_j-correlated part, $T_{i,j}W_{i,j}^T$, a Y_j-uncorrelated part, $\mathbf{T}_{i,j,O}\mathbf{P}_{i,j,O}^T$, and the unmodeled variance $\mathbf{E}$ as

$$\mathbf{X}_i = T_{i,j}W_{i,j}^T + \mathbf{T}_{i,j,O}\mathbf{P}_{i,j,O}^T + \mathbf{E}_{i,j},$$

and new regressor matrices $\mathbf{X}_{\text{Top},j}$ for each trait j are formed by concatenation:

$$\mathbf{X}_{\text{Top},j} = [T_{1,j}W_{1,j}^T + \mathbf{E}_{1,j}; \ldots ; T_{n,j}W_{n,j}^T + \mathbf{E}_{n,j}].$$

Top-level models are then estimated by ordinary OPLS regression between $\mathbf{X}_{\text{Top},j}$ and Y_j. MB-OPLS for a single block is equivalent to ordinary OPLS. With this definition, MB-OPLS for a single block is equivalent to ordinary OPLS. MB-OPLS shares characteristics with the previously proposed MB-PLS [1] but gives equal emphasis to each metabolite instead of each block.

1.2 Miscellaneous

All data analysis was carried out using the R statistical programming environment v2.12.1 [2].

The ANOVA for testing for significantly different metabolite abundances was done by pairwise comparisons of Indica I (I_1), Indica II (I_2) and Japonica (J) populations using the LIMMA package [3]. Setting the abundance for metabolite i to

$$m_i = I_{1i} + I_{2i} + J_i + \epsilon$$

and then calculating the contrasts I1-I-2, I1-J and I2-J.

Word clouds were generated by uploading color and correlation loadings to `http://www.wordle.net`.

The Mantel test [4] was performed using the ade4 package [5].

1.3 Chemical diversity

Chemical diversity was estimated by fetching predicted physicochemical properties from the ChemSpider database (`http://www.chemspider.com`) for the detected metabolites and the metabolites mentioned in the RiceCyc database. Vapor pressure was log transformed and all traits were scaled to unit variance to give them equal importance. Chemical coverage was defined as the percentage of variance among the PlantCyc metabolites that could be predicted using a principal component analysis (PCA) model of the properties of the detected metabolites. See ref. [6] for details.

2 Metabolomics metadata

Based on the suggestion of the Metabolomics Standards Initiative [7], metadata in this study was shown as follows.

2.1 Plant context metadata

2.1.1 BioSource Species

Oriza sativa L.

2.1.2 Genotype

The rice diversity research set (RDRS) consists of 68 accessions and Nipponbare and Kasalath as reference cultivars [8]. We furthermore chose to include the salinity resistant cultivar Pokkari. Four additional varieties outside the RDRS (Yumetoiro, Hoshiyutaka, Kinmaze and Soft158) and two amylose hyper accumulating *Starch synthase IIIa* (*SSIIIa*) knock-out lines (*Tos17* retro-transposon insert): *e1*, a single knock-out (Nipponbare background) [9] and *4019*, double knock-out, with Nipponbare/Kinmaze backgroundwere used for the validation experiment.

2.1.3 Organ

Kernel

2.1.4 Organ specification

Brown rice

2.1.5 Growth condition

Twenty-five rice seeds for each of RDRS were sown at a rice field in NIAS, Tsukuba (Lat., 36.030753; Long. 140.099858), Japan in Spring. For the external set of samples, seeds were grown at a rice field in Akita (Lat. 39.803897; Long. 140.046451), Japan.

2.1.6 Sampling and sampling date

For RDRS, seeds were harvested independently for each variety after 40 days, starting from the day on which the first panicle of rice was observed in 2005 and 2006. For others, seeds were also harvested independently for each variety in 2005 (for Yumetoiro, Hoshiyutaka, Kinmaze, Soft158, *el* and Nipponbare) and 2008 (for *4019* and Nipponbare).

2.2 Chemical analysis metadata

2.2.1 BioSource amount

For RDRS and Hoshiyutaka, 100 seeds of each variety were selected according to the average weight and length of seeds. After separating the husks from the seeds, the brown rice seeds obtained were bulked and crushed by using a Retsch mixer mill MM301 at a frequency of 20 Hz for 2 min at 4 °C. Successively, the obtained powder was divided into three to four pools. For external set of samples harvested in Akita, 100 seeds of each biological replicate were selected and crushed in the same way as RDRS.

2.2.2 Sample processing and extraction

Extraction and derivatization for GC-MS One hundred milligrams of each sample was extracted with extraction buffer [methanol/chloroform/water (3:1:1, v/v/v)] at a concentration of 100 mg/ml containing 10 stable isotope reference compounds as follows:

- [$^{2}H_{4}$]-succinic acid,
- [$^{13}C_{5}$,^{15}N]-glutamic acid,
- [$^{2}H_{7}$]-cholesterol,
- [$^{13}C_{3}$]-myristic acid,
- [$^{13}C_{5}$]-proline,
- [$^{13}C_{12}$]-sucrose,
- [$^{13}C_{4}$]-hexadecanoic acid,
- [$^{2}H_{4}$]-1,4-butanediamine,
- [$^{2}H_{6}$]-2-hydoxybenzoic acid and
- [$^{13}C_{6}$]-glucose.

Each isotope compound was adjusted to a final concentration of 15 ng/μl for each 1-μl injection. After centrifugation, a 200-μl aliquot of the supernatant (ca. 25 mg of each sample) was drawn and transferred into a glass insert vial. The extracts were evaporated to dryness in an SPD2010 SpeedVac® concentrator from ThermoSavant (Thermo electron corporation, Waltham, MA, USA). For methoximation, 30 μl of methoxyamine hydrochloride (20 mg/ml in pyridine) was added to the sample. After 24 h of derivatization at room temperature, the sample was trimethylsilylated for 1 h using 30 μl of MSTFA with 1% TMCS at 37°C with shaking. Thirty μl of n-heptane was added following silylation. All the derivatization steps were performed in the vacuum glove box VSC-100 (Sanplatec, Japan) filled with 99.9995% (G3 grade) of dry nitrogen.

For methoximation, 30 μl of methoxyamine hydrochloride (20 mg ml^{-1} in pyridine) was added to the sample. After 24 h of derivatization at room temperature, the sample was trimethylsilylated for 1 h using 30 μl of MSTFA with 1% TMCS at 37°C with shaking. Thirty μl of n-heptane was added following silylation. All the derivatization steps were performed in the vacuum glove box VSC-100 (Sanplatec, Japan) filled with 99.9995% (G3 grade) of dry nitrogen.

Extraction for LC-MS 100 mg of each sample was extracted with extraction buffer [methanol/water (5:95, v/v)] at a concentration of of 100 mg/ml using a Retsch mixer mill MM310 at a frequency of 20 Hz for 10 min at 4°C. After centrifugation for 10 min at 15,000 × g, 500 μl of the supernatant was transferred into a tube and diluted in 0.1% acetic acid solution. and then it was filtered using an Oasis® HLB μ-elusion plate (30 μm, Waters Co., Massachusetts, USA). The extracts ca. 0.1 mg of each sample) were evaporated to dryness in an SPD2010 SpeedVac® concentrator.The extracts were dissolved by 200 μl of water containing five reference compounds as follows:

- 0.5 mg/l of lidocaine,
- 1.0 mg/l of ampiciline,
- 1.0 mg/l of torperizone,
- 0.5 mg/l of 10-camphor sulfonic acid and
- 1.0 mg/l of 2-naphthalene-4-sodium sulfate.

Extraction for CE-MS 50 mg of each sample was extracted in 20 volumes of methanol containing 8μM of two reference compounds (methionine sulfone for cation and camphor 10-sulfonic acid for anion analyses) using a Retsch mixer mill MM310 at a frequency of 27 Hz for 1 min. The extracts were then centrifuged at 20,400 × g for 3 min at 4 °C. Five hundred-μl aliquot of the supernatant was transferred into a tube. Five hundred μl of chloroform and 200 μl of water was added into the tube to perform liquid-liquid distribution. The upper layer was evaporated for 30 min at 45°C by a centrifugal concentrator to obtain two layers. For removing high-molecular-weight compounds such as oligo-sugars, the upper layer was centrifugally filtered through a Millipore 5-kDa cutoff filter at 9,100 g for 120 min at 4°C. The filtrate was dried for 120 min by a centrifugal concentrator. The residue (ca. 25 mg of each sample) was dissolved into 20 μl of water containing 200 μM of internal standards (3-aminopyrrolidine for cation and trimesic acid for anion analyses) that were used for compensation of migration time in the peak annotation step.

Extraction for IT-MS Each Sample (50 mg) was extracted with 750 μl of chloroform/MeOH (1:1, v/v) containing 1.25 μM 1,2-dioctanoyl-sn-glycero-3-phosphocholine (SIGMA) followed by centrifugation at 10,000g at 4°C for 5 min. The supernatant was transferred to a 2 ml tube, and the extraction procedure was repeated again. The combined supernatant was evaporated to dryness by SPD2010 SpeedVac® concentrator. The residue was dissolved in 750 μl of ethanol, and centrifuged at 10,000g at 4°C for 5 min. Six hundred microlitter of the supernatant was transferred to a glass tube for polar-lipid analysis.

2.2.3 MS conditions

GC-TOF-MS conditions One microliter of extracts (ca. 277.8μg each sample) was injected in the splitless mode by an CTC CombiPAL autosampler (CTC analytics, Zwin-gen, Switzerland) into an Agilent 6890N gas chromatograph (Agilent Technologies, Wilmingston, USA) equipped with a 30 m × 0.25 mm inner diameter fused-silica capillary column with a chemically bound 0.25-μl film Rtx-5 Sil MS stationary phase (RESTEK, Bellefonte, USA) for metabolome analysis.

Helium was used as the carrier gas at a constant flow rate of 1 ml min^{-1}. The temperature program for metabolome analysis started with a 2-min isothermal step at 80 °C and this was followed by temperature ramping at 30 °C to a final temperature of 320 °C, which was maintained for 3.5 min. The transfer line and the ion source temperatures were 250 and 200 °C, respectively. Ions were generated by a 70-eV electron beam at an ionization current of 2.0 mA. The acceleration voltage was turned on after a solvent delay of 222 and 237 s. Data acquisition was performed on a Pegasus III and Pegasus IV TOF mass spectrometers (LECO, St. Joseph, MI, USA) with an acquisition rate of 30 spectra s^{-1} in the mass range of a mass-to-charge ratio of m/z = 60–800.

Alkane standard mixtures (C8–C20 and C21–C40) were purchased from Sigma–Aldrich (Tokyo, Japan) and were used for calculating the retention index (RI) [10, 11]. The normalized response for the calculation of the signal intensity of each metabolite from the mass-detector response was obtained by each selected ion current that was unique in each metabolite MS spectrum to normalize the peak response. For quality control, we injected methylstearate in every 6 samples. Data was normalized using the CCMN algorithm [12].

LC-q-TOF-MS conditions After filtration of the extracts (Ultrafree-MC, 0.2 μm pore size; Millipore), 5 μl of extracts (ca. 0.1 mg each sample) was analyzed using an LC-MS system equipped with an electrospray ionization (ESI) interface (HPLC, Waters Acquity UPLC system; MS, Waters Q-Tof Premier). The analytical conditions were as follows. HPLC: column, Acquity bridged ethyl hybrid (BEH) C18 (pore size 1.7 μ m, length 2.0 × 100 mm, Waters); solvent system, acetonitrile (0.1% formic acid):water (0.1% formic acid); gradient program, 1 : 99 v/v at 0 min, 1 : 99 v/v at 0.1 min, 64.0 : 0.5 at 10.0 min, 99.5 : 0.5 at 11.5 min, 1 : 99 v/v at 11.6 min and 1 : 99 at 14.0 min; flow rate, 0.3 ml min^{-1}; temperature, 38°C; MS detection: capillary voltage, +3.0 keV; cone voltage, 23 V for positive mode and 35 V for negative mode; source temperature, 120°C; desolvation temperature, 450°C; cone gas flow, 50 l h^{-1}; desolvation gas flow, 800 l/ h; collision

energy, 2 V for positive mode and 5 V for negative mode ; detection mode, scan (m/z 100–2000; dwell time 0.45 sec; interscan delay 0.05 sec, centroid). The scans were repeated for 14.0 min in a single run. The data were recorded using MassLynx version 4.1 software (Waters).

CE-TOF-MS conditions All CE-TOFMS experiments were performed using an Agilent CE capillary electrophoresis system (Agilent Technologies, Waldbronn, Germany), an Agilent G3250AA LC/MSD TOF system (Agilent Technologies, Palo Alto, CA), an Agilent 1100 series binary HPLC pump, and the G1603A Agilent CE-MS adapter and G1607A Agilent CE-ESI-MS sprayer kit. The G2201AA Agilent ChemStation software for CE and the Analyst QS software for TOFMS were used.

Separation column and electrolytes: Separations were carried out using a fused silica capillary (50 μm i.d. x 100 cm total length) filled with 1 M formic acid for cation analyses or with 20 mM ammonium formate (pH 10.0) for anion analyses as the electrolyte. The capillary temperature was maintained at 20 °C.

Sample injection: The sample solutions (11.25 μl of extracts, ca. 0.6 μg of each sample) were injected at 50 mbar for 15 sec (15 nl). The sample tray was cooled below 4 °C.

Separation parameters: Prior to each run the capillary was flushed with electrolyte for 5 min. The applied voltage for separation was set at 30 kV. Fifty percent (v/v) methanol/water containing 0.5 μM reserpine was delivered as the sheath liquid at 10 μL/min.

Ionization: ESI-TOFMS was conducted in the positive ion mode for cation analyses or in the negative ion mode for anion analyses, and the capillary voltage was set at 4 kV.

Dry gas condition: A flow rate of heated dry nitrogen gas (heater temperature 300 °C) was maintained at 10 psig.

Voltage settings in TOF-MS: The fragmentor, skimmer, and Oct RFV voltage were set at 110V, 50V, and 160V for cation analyses or at 120V, 60V, and 220V for anion analyses, respectively.

Mass calibration: Automatic recalibration of each acquired spectrum was performed using reference masses of reference standards. The methanol dimer ion ($[2M+H]^+$, m/z = 65.0597) and reserpine ([M+H]+, m/z = 609.2806) for cation analyses or the formic acid dimer ion ([2M-H]-, m/z = 91.0037) and reserpine ($[M-H]^-$, m/z = 607.2661) for anion analyses provided the lock mass for exact mass measurements.

Mass data acquirement: Exact mass data were acquired at a rate of 1.5 cycles/sec over a 50-1000 m/z range.

Quality control: In an every single sequence analysis (maximum 36 samples) on our CE-MS system, we analyzed the standard compound mixture at the first and the end of sample analyses. The detected peak area of standard compound mixture was checked in point of reproducible sensitivity. Standard compound mixture composed of major detectable metabolites including amino acids and organic acids, and this mixture was newly prepared at least once a half year. In all analyses in this study, there were no differences in the sensitivity of standard compounds mixture.

LC-IT-TOF-MS conditions Extracts (0.5 μl, ca. 33.3 μg of each sample) was analyzed by LC-MS with ESI interface (LC, Shimadzu LC-20AD system; MS, Shimadzu LCMS-IT-TOF) operated by Shimadzu LCMSsolution software (version 3.60). Two-solvent system was used for separation of each metabolite. The analytical conditions were as follows. Column, Shim-pack XR-ODS (2.0 mm I.D., 50 mm long); solvent A, water (1% 1M ammonium formate and 0.1% formic acid); solvent B, acetonitrile/isopropyl alcohol (40:60, v/v. 1% 1M ammonium formate and 0.1% formic acid); gradient program, 40% B at 0 min, 75% B at 3 min, 95% B at 10 min, 100% B at 19 min, 100% B at 27 min, 40% B at 27.01 min (total run time, 30 min); flow rate, 0.3 ml/min; column temperature, 55˚C; MS interface voltage, 4.50 kV, nebulizer gas, 1.50 L/min; CDL temperature200.0˚C, heat block temperature, 200˚C; detection mode, scan (m/z 150˜1600, positive); scan time, 0.25 sec; ion accumulation time, 20 msec.

2.2.4 Data processing

GC-MS Nonprocessed MS data from GC-TOF/MS analysis were exported in NetCDF format generated by chromatography processing and mass spectral deconvolutionsoftware, Leco ChromaTOF version 2.32 and 3.22 (LECO, St. Joseph, MI, USA) to MATLAB 6.5 and 7.0 (Mathworks, Natick, MA, USA), where all data-pretreatment procedures, such as smoothing, alignment, time-window setting, and H-MCR, were carried out [13]. The resolved MS spectra were matched against reference mass spectra using the NIST mass spectral search program for the NIST/EPA/NIH mass spectral library (version 2.0) and our custom software for peak annotation written in JAVA. Peaks were identified or annotated based on RIs and the reference mass spectra comparison to the Golm Metabolome Database (GMD) released from CSB.DB[1] [14]] and our in-house spectral library. The metabolites were identified by comparison with RIs from the library databases (GMD and our own library) and with those of authentic standards, and the metabolites were defined as annotated metabolites on comparison with mass spectra and RIs from these two libraries.

LC-MS The profiling data files recorded in the MassLynx format (raw) were converted to the NetCDF format using the DataBridge function of MassLynx 4.1. From the set of NetCDF data files, the data matrix was generated using the MetAlign software (De Vos et al., 2007). By using this procedure, the data matrixes with unit mass data were generated. The data matrices were processed using in-house software written in Perl/Tk. The original peak intensity values were divided with that of the internal standards (lidocaine at m/z 235 [M + H]+ and (–)-camphor-10-sulfonic acid at m/z 231 [M – H]– for the positive and negative ion modes, respectively) determined in the same samples to normalize the peak intensity values among the metabolic profile data.

CE-MS An original data file (.wiff) was converted to an unique binary file (.kiff) using in-house software (nondisclosure). Peak picking and alignment were performed using the another in-house software (nondisclosure), peaks were picked and aligned among samples automatically. By contrast with the detected m/z and migration time values of standard compounds including internal standards, peaks were annotated automatically using the same software. For normalization, the individual area of the detected peaks was divided by the peak area of the internal reference standards. Based on the calibration curves for standard compounds, peak area values were converted into values corresponding to amounts.

IT-MS Peak picking and peak alignment were performed by Profiling Solution (version 1.0.76.0) (Shimadzu, Kyoto, Japan) from original data file (.lcd). The parameters of Profiling Solution were as follows: ion m/z tolerance, 20 mDa; Ion RT tolerance, 0.1 min; Ion intensity threshold, 2e4; detect isomer valley, 20%; allow some ion without isotope peak, ON; time range for processing, 0–20 min. The data matrix exported from Profiling Solution was normalized based on the intensity of [M+H]+ of 1,2-dioctanoyl-sn-glycero-3-phosphocholine. Peak height of individual lipid molecules were calculated based on the m/z values of their molecular-related ions or fragment ions.

[1]http://csbdb.mpimp-golm.mpg.de/csbdb/gmd/msri/gmd_msri.html

Annotation process was performed based on theoretical m/z values of each possible glycerolipid species in plants using in-house Perl script.

Supplementary References

[1] JA. Westerhuis, T. Kourti, and JF. MacGregor. Analysis of multiblock and hierarchical PCA and PLS models. *J Chemom*, 12:301–321, 1998.

[2] R Development Core Team. *R: A language and environment for statistical computing.* R Foundation for Statistical Computing, Vienna, Austria, 2004.

[3] R. Gentleman, V. Carey, S. Dudoit, R. Irizarry, W. Huber, and G.K. Smyth, editors. *LIMMA: linear models for microarray data. In: Bioinformatics and computational biology solutions using R and Bioconductor*, chapter 23. Springer, New York, 2005.

[4] N Mantel. The detection of disease clustering and a generalized regression approach. *Cancer Res*, 27:209–220, 1967.

[5] S. Dray and A.B. Dufour. The ade4 package: implementing the duality diagram for ecologists. *J Stat Softw*, 22:1–20, 2007.

[6] Miyako Kusano, Henning Redestig, Tadayoshi Hirai, Akira Oikawa, Fumio Matsuda, Atsushi Fukushima, Masanori Arita, Shin Watanabe, Megumu Yano, Kyoko Hiwasa-Tanas, Hiroshi Ezura, and Kazuki Saito. Covering chemical diversity of genetically-modified tomatoes using metabolomics for objective substantial equivalence assessment. *PLoS ONE*, 6:e16989, February 2011.

[7] Preeti Bais, Stephanie M Moon, Kun He, Ricardo Leitao, Kate Dreher, Tom Walk, Yves Sucaet, Lenore Barkan, Gert Wohlgemuth, Mary R Roth, Eve Syrkin Wurtele, Philip Dixon, Oliver Fiehn, B. Markus Lange, Vladimir Shulaev, Lloyd W Sumner, Ruth Welti, Basil J Nikolau, Seung Y Rhee, and Julie A Dickerson. Plantmetabolomics.org: a web portal for plant metabolomics experiments. *Plant Physiol*, 152(4):1807–1816, Apr 2010.

[8] Yoichiro Kojima, Kaworu Ebana, Kaworu Ebana, Shuichi Fukuoka, Tsukasa Nagamine, and Makoto Kawase. Development of an RFLP-based rice diversity research set of germplasm. *Breeding Science*, 55:431–440, 2005.

[9] Naoko Fujita, Mayumi Yoshida, Tomonori Kondo, Kaori Saito, Yoshinori Utsumi, Takashi Tokunaga, Aiko Nishi, Hikaru Satoh, Jin-Hee Park, Jay-Lin Jane, Akio Miyao, Hirohiko Hirochika, and Yasunori Nakamura. Characterization of SSIIIa-deficient mutants of rice: the function of SSIIIa and pleiotropic effects by SSIIIa deficiency in the rice endosperm. *Plant Physiol*, 144(4):2009–2023, Aug 2007.

[10] Nicolas Schauer, Dirk Steinhauser, Sergej Strelkov, Dietmar Schomburg, Gordon Allison, Thomas Moritz, Krister Lundgren, Ute Roessner-Tunali, Megan G Forbes, Lothar Willmitzer, Alisdair R Fernie, and Joachim Kopka. GC-MS libraries for the rapid identification of metabolites in complex biological samples. *FEBS Lett*, 579(6):1332–1337, Feb 2005.

[11] Cornelia Wagner, Michael Sefkow, and Joachim Kopka. Construction and application of a mass spectral and retention time index database generated from plant GC/EI-TOF-MS metabolite profiles. *Phytochemistry*, 62(6):887–900, Mar 2003.

[12] Henning Redestig, Atsushi Fukushima, Hans Stenlund, Thomas Moritz, Masanori Arita, Kazuki Saito, and Miyako Kusano. Compensation for systematic cross-contribution improves normalization of mass spectrometry based metabolomics data. *Anal Chem*, 81:7974–7980, 2009.

[13] Pär Jonsson, Annika I Johansson, Jonas Gullberg, Johan Trygg, Jiye A, Bjørn Grung, Stefan Marklund, Michael Sjöström, Henrik Antti, and Thomas Moritz. High-throughput data analysis for detecting and identifying differences between samples in GC/MS-based metabolomic analyses. *Anal Chem*, 77(17):5635–5642, Sep 2005.

[14] Joachim Kopka, Nicolas Schauer, Stephan Krueger, Claudia Birkemeyer, Björn Usadel, Eveline Bergmüller, Peter Dörmann, Wolfram Weckwerth, Yves Gibon, Mark Stitt, Lothar Willmitzer, Alisdair R Fernie, and Dirk Steinhauser. GMD@CSB.DB: the Golm Metabolome Database. *Bioinformatics*, 21(8):1635–1638, Apr 2005.

Supplementary Table S1: The examined quality traits. NIAS: National Institute for Agricultural Sciences, http://www.gene.affrc.go.jp/databases-core_collections_wr_en.php. JFRL: Japan Food Research Laboratories

Trait	Unit	Assay	Source
Ear emergence day	Days after sowing	Observation	NIAS
Amylose ratio (content)	Percent	Iodine calorimetry	NIAS
Kernel length	mm	Micro-meter	NIAS
Kernel width	mm	Micro-meter	NIAS
Kernel size	mm x mm	Kernel length x kernel width	NIAS
Rice hull width	mm	Micro-meter	NIAS
Rice hull length	mm	Micro-meter	NIAS
Riboflavin	mg per 100g of kernels	quantitative analysis	JFRL
α-tocopherol	mg per 100g of kernels	quantitative analysis	JFRL
γ-tocopherol	mg per 100g of kernels	quantitative analysis	JFRL
Lysine	mg per 100g of kernels	quantitative analysis	JFRL
Culm length	mm	Micro-meter	NIAS
1000 Kernel weight	g	Weight of 10 kernels times 100	NIAS
Germination rate	Percent	Observation	NIAS
Iron content	mg per 100g of kernels	quantitative analysis	JFRL
Zinc content	mg per 100g of kernels	quantitative analysis	JFRL
Ear number	Count		NIAS

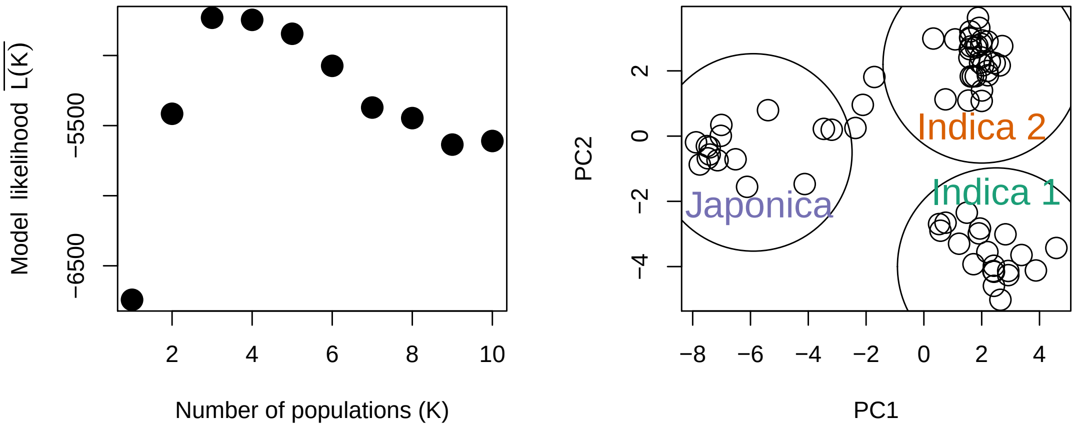

Supplementary Figure S1: The number of populations. The WRC is genetically heterogenic and this is seen in the RFLP data. (a) STRUCTURE was used to estimate the number of sub-populations, K. The optimal number in terms of log-likelihood appears to be 3. (b) Principal coordinates plot of the RFLP data of the RDRS. The three populations corresponding to two varieties of Indica rice and one Japonica are clearly visible.

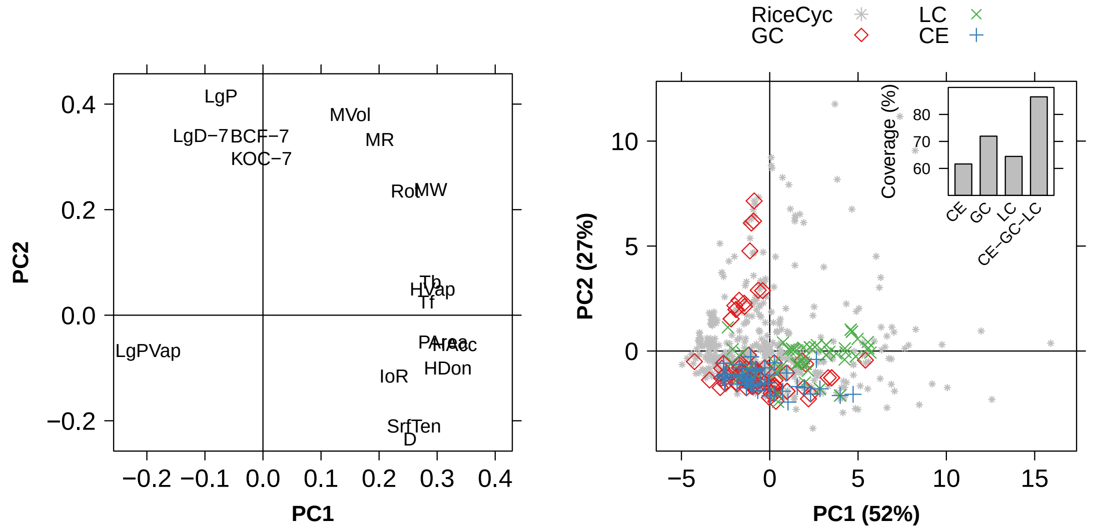

Supplementary Figure S2: Evaluation of the achieved coverage. PCA was performed on the predicted physicochemical properties of the detected metabolites and the metabolites in the RiceCyc database. (a) The loading plots show that PC1 is dominated by size-related- and PC2 by solubility-related properties. (b) The score plots show that the distribution of the detected metabolites occupies a similar space as the reference metabolites. The inset barplot shows the ratio of variance among the reference metabolites covered by each of the individual platforms and the summarized data set. Abbreviations: Log vapor pressure (LgPVap), octanol:water partitioning coefficient (LgP), octanol:water solubility distribution coefficient at pH 7.4 (LgD-7), biological concentration factor at pH 7.4 (BCF-7), adsorption coefficient at pH 7.4 (KOC), molecular volume (MVol), molecular refractivity (MR), molecular weight (MW), free rotating bonds (Rot), boiling temperature (Tb), flash point (Tf), enthalpy of vaporization (HVap), polar surface area (PArea), number of H-bond donors/acceptors (HDon/HAcc), surface tension (SrfTen), density (D), index of refraction (IoR).

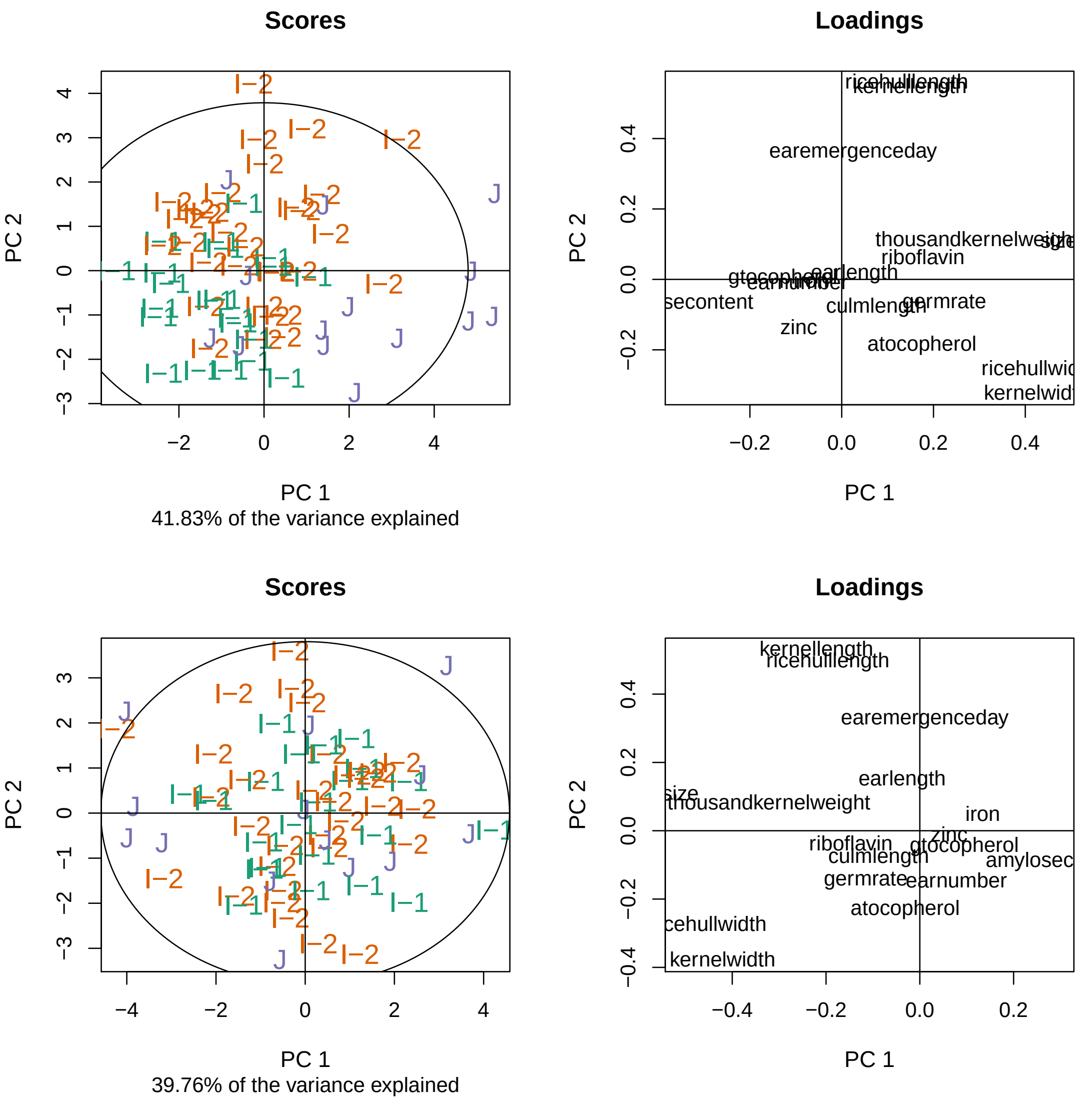

Supplementary Figure S3: (a) PCA of the quality trait data showing how the genetic sub-populations differ from each other in terms of their quality traits. (b) PCA of the quality trait data after correction for the genetic sub-populations.

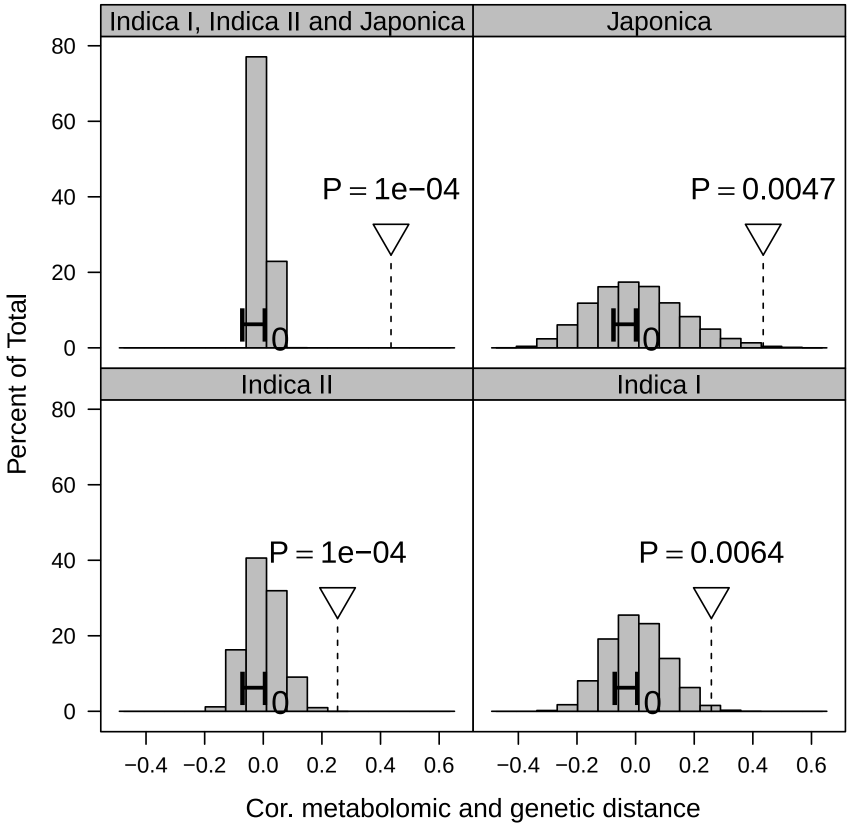

Supplementary Figure S4: Mantel test of the correlation between metabolomic and genetic distances. The observed overall correlations between genetic (c.f. Fig. 2a) and metabolomic distances (c.f. Fig. 2c) are higher than after data randomization (H_0) for both the whole RDRS and for the three sub-populations individually.

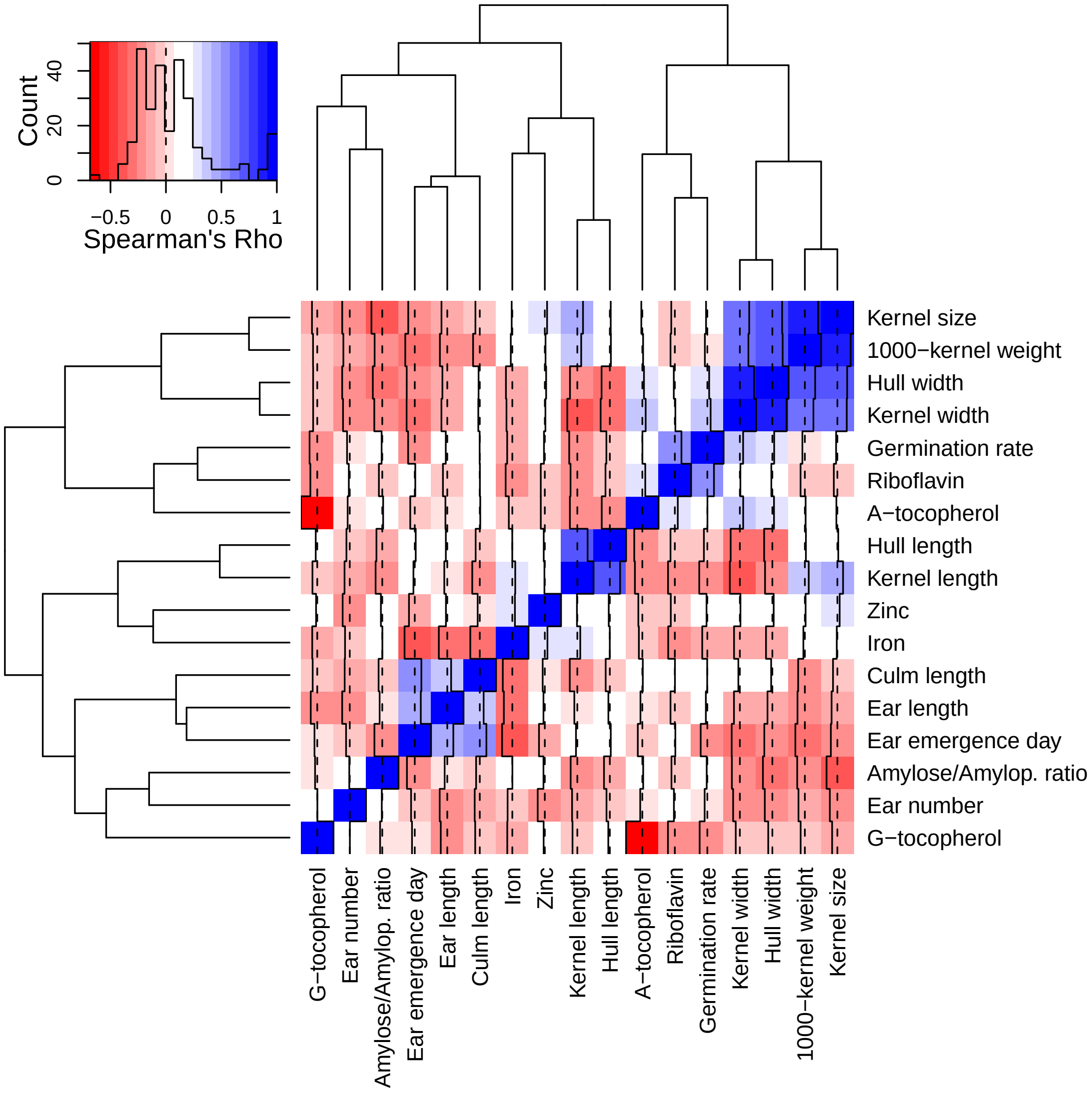

Supplementary Figure S5: Correlation heatmap of the population structure corrected quality trait data.

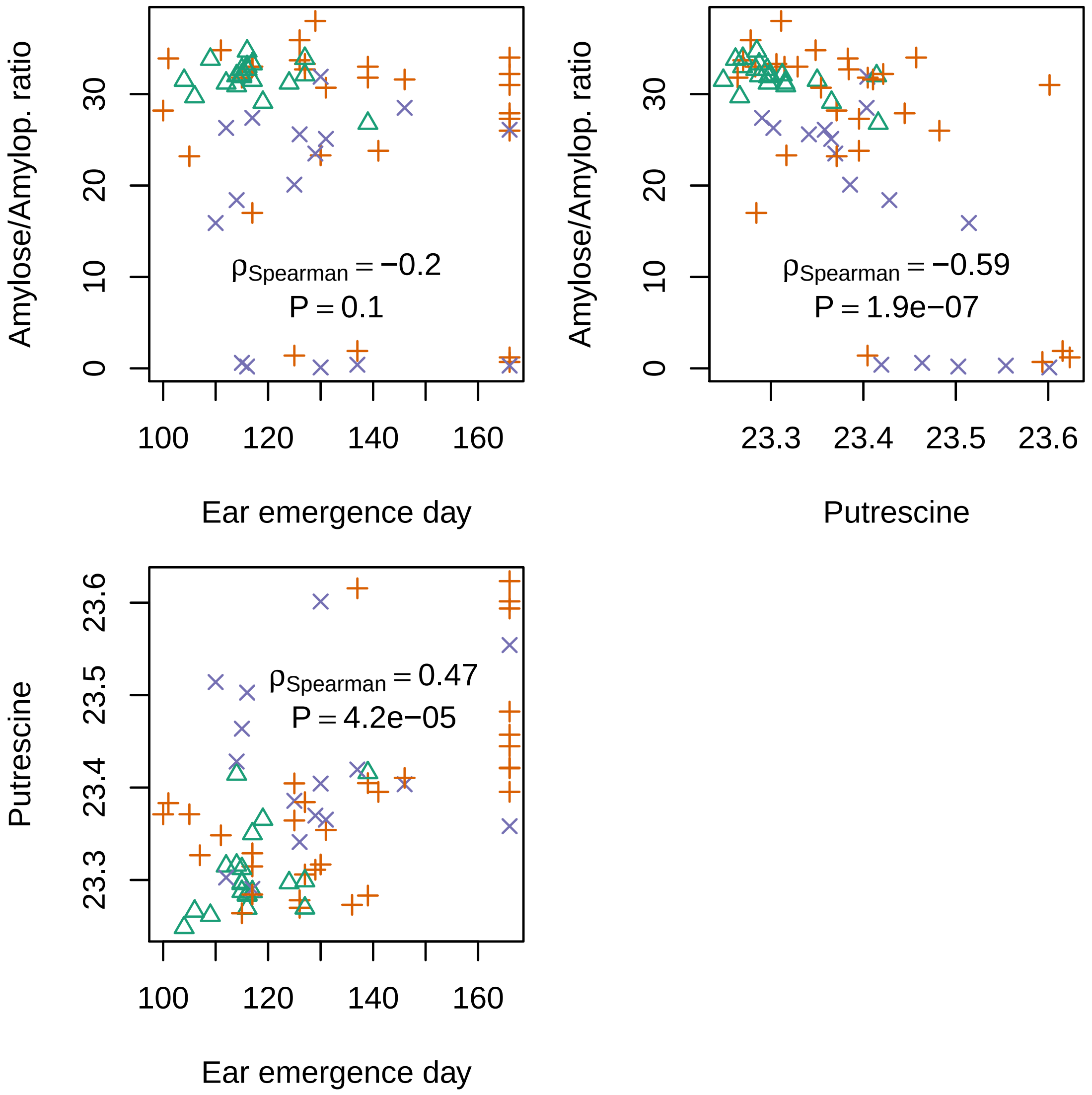

Supplementary Figure S6: Scatter plots of putrescine abundance versus the ear emergence day and amylose/amylopectin ratio. Putrescine is correlated to both of these traits despite the traits not being prominently correlated to each other.
