# Supplementary figures and images for: Exploring molecular backgrounds of quality traits in rice by predictive models based on high-coverage metabolomics
Source: BMC Syst Biol. 2011 Oct 28;5:176. doi: 10.1186/1752-0509-5-176 (PMC3305925; doi:10.1186/1752-0509-5-176)

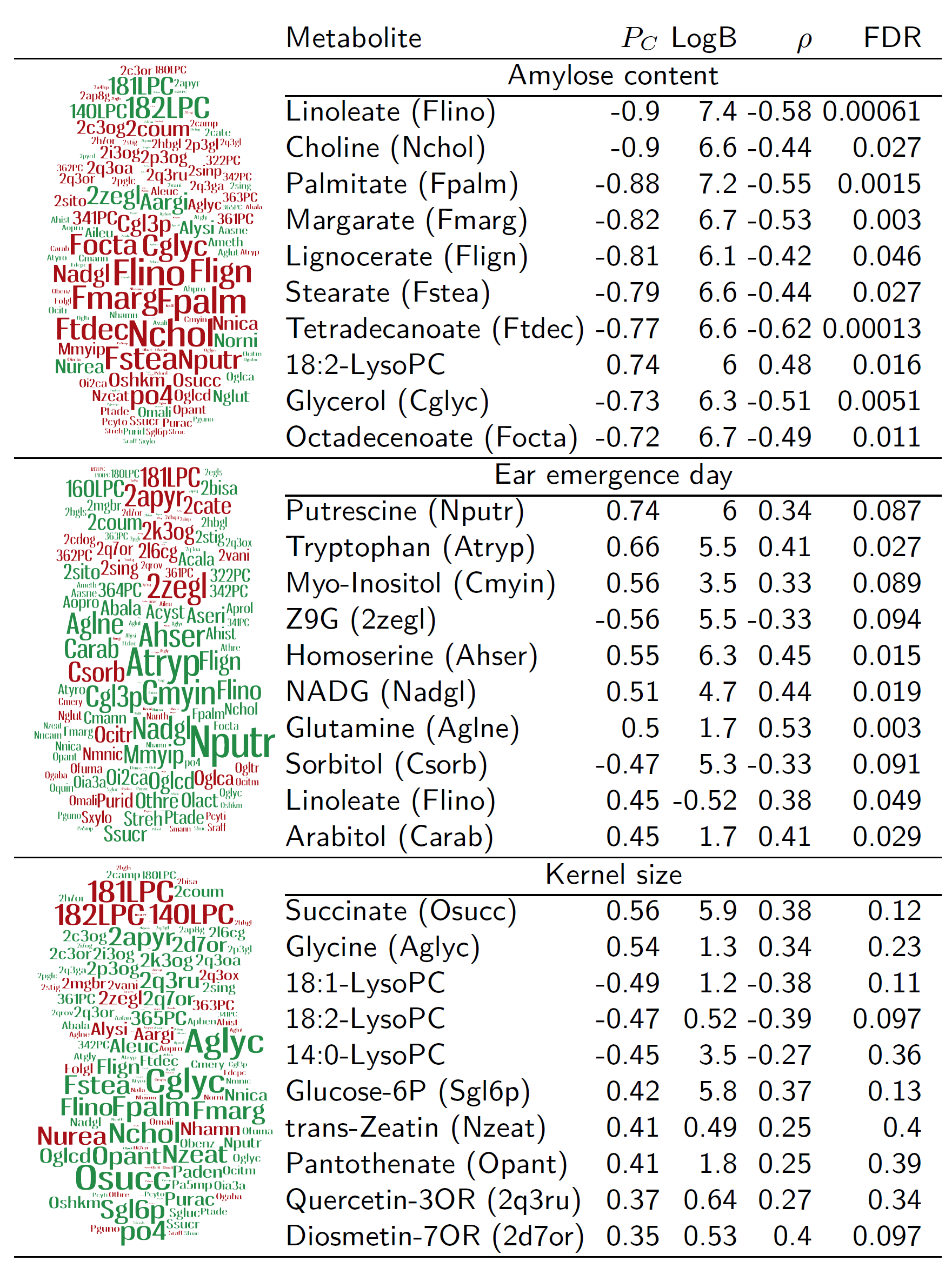

Supplement: Additional file 3 — Influential metabolites. Correlation loading, PC, indicate proximity between the metabolite and the trait-correlated variance. log B indicates how many times more likely the alternative hypothesis (actual association between trait and metabolite) is than the null-hypothesis (no association). Spearman's correlation ρS with associated FDR indicates the direct bivariate correlation. Word clouds are ordered alphabetically and have font sizes proportional to the corresponding correlation loading (PC). Green and red indicate apositive and negative correlation with the trait, respectively. The spatial layout is abitrary. Where present, initial capital letters of the metabolite abbreviations indicate type of molecule (F, fatty acid; C, alcohol; P, purine/pyrimidine; S, sugar; N, nitrogen containing; A, amino acid; 2, secondary metabolite) [file 1752-0509-5-176-S3.png]
